# Male involvement and associated factors in birth preparedness and complication readiness in Debre Berhan Town, North East Ethiopia

**DOI:** 10.11604/pamj.2020.35.36.10346

**Published:** 2020-02-10

**Authors:** Melkamu Worku, Berhanu Boru, Abdella Amano, Abdulbasit Musa

**Affiliations:** 1 Department of Midwifery, College of Medicine and Health Sciences, Wolaita Sodo University, Wolaita Sodo, Ethiopia; 2 Department of Midwifery, College of Medicine and Health Sciences, University of Gondar, Gondar, Ethiopia; 3 Department of Biostatistics and Epidemiology, School of Public health, Hawassa University, Hawassa, Ethiopia; 4 Departments of Midwifery, College of Health and Medical Sciences, Haramaya University, Haramaya, Ethiopia

**Keywords:** Birth preparedness and complication readiness, Debre Berhan town, Ethiopia male involvement

## Abstract

**Introduction:**

Men play indispensable role in health and wellbeing of mothers and children. Their level of involvement with regards to birth preparedness and complication readiness is understudied. This study was therefore carried out to assess male involvement and associated factors in birth preparedness and complication readiness in Debre Berhan town, North East Ethiopia.

**Methods:**

Community based cross-sectional study was conducted from July 1^st^ - 30^th^, 2014 in Debre Berhan town among 806 study participants. Cluster sampling technique was employed to select study participants. Data were collected using a structured and pre-tested questionnaire by face-to-face interview technique. Bivariate and multivariate analyses were performed to check associations and control confounding.

**Results:**

The study showed that male involvement in birth preparedness and complication readiness found to be 51.4%. Educational status of tertiary level (AOR = 4.37 95% (CI: 2.10, 9.13), having 1 or 2 children (AOR = 2.52, 95% CI:1.30,4.87) and 3 or 4 (AOR = 5.19, 95% CI:2.44,11.03), knowledge of danger signs (AOR = 7.71, 95% (CI:5.15, 11.54), knowledge of birth preparedness and complication readiness (AOR = 11.98, 95% CI:7.73,18.56) and attitude (AOR = 2.23, 95% CI: 1.41,3.51) were significantly associated with male involvement in birth preparedness and complication readiness.

**Conclusion:**

Male involvement in birth preparedness and complication readiness found to be low in study area. Education, number of children, knowledge on danger signs, knowledge on birth preparedness and complication readiness and attitude were factors associated with male involvement. Creating awareness on danger signs of pregnancy, birth preparedness and complication readiness both at community and institutional level were recommended in order to increase male involvement in birth preparedness and complication readiness.

## Introduction

Maternal mortality has continued to be a major challenge to health systems worldwide [1, 2]. Although there is substantial decline in maternal deaths and global progress to decrease maternal mortality ration (MMR), the ratios were much higher throughout sub-Saharan Africa than in other regions [3, 4]. According to Maternal Mortality Estimation Inter-Agency Group (MMEIG) report of 2013, sub-Saharan Africa had the highest MMR at 510 maternal deaths per 100,000 live births while global MMR is 210 maternal deaths per 100,000 live births. Additionally, Ethiopia is one of ten countries which account for 58% of global maternal deaths with maternal mortality ratio of 420 [3]. These deaths occur due to pregnancy, childbirth or postpartum problems; and an important strategy that can decrease the number of women dying from such problems is making a birth plan that constitutes birth preparedness and complication readiness (BP/CR) measures for pregnant women, their husbands or partners and other family members [5]. BP/CR cannot be achieved with the effort of single individual or party. It needs the involvement of the pregnant women and her partners, communities, facility setup, service providers and policy makers [6, 7]. As one of the key component of BP/CR men’s involvement in the area is strongly recommended by international conference for population development (ICPD) as emphasis should be made on men’s shared responsibility and to promote their active involvement in maternity care [8]. However, little is known about the current level of male involvement and associated factors on birth preparedness and complication readiness in the study areas. Hence, assessing the factors affecting male involvement in birth preparedness and complication readiness in the study area is very important to decrease delay to seek health care and thereby reduce maternal and infant deaths.

## Methods

A community based cross sectional study design was conducted in Debre Berhan town from July 1^st^ – 30^th^, 2014. The town is located in the North Shoa zone of the Amhara Region; 130km from the capital city of Ethiopia, Addis Ababa in the North East direction and at 795km from the capital city of the region (Bahir Dar). Administratively, Debre Berhan town is divided into 9 kebeles and an estimated population size of 65,214 [9]. All husbands whose wives were pregnant or had children below one year old were included in the study. Cluster sampling technique was employed. From the nine kebeles five were selected randomly using lottery method. Then all eligible male in the selected cluster were included in the study. Sample size was calculated by using single population proportion formula with assumptions of desired precision (d) = 5%, expected prevalence (p): male involvement in BP/CR taken as 50%, confidence level taken as 95%, which means α set at 0.05 and Z_α/2_ = 1.96 (value of Z at α = 0.05 or critical value for normal distribution at 95% CI). Hence, the calculated sample size was = 384. Since the sampling technique was cluster, design effect of 2 was considered, for possible none response rate 10% and the final sample size was 845. The data was collected by using pretested, structured and interviewer administered questionnaires. The questionnaires were administered by five BSc midwives. They had two days of training including the supervisors for the purposes of data collection. The data collection process was closely supervised by one field supervisor who has Bsc degree in public health and the principal investigator. In this study male involvement in birth preparedness and complication readiness was measured based on seven indicators. Thus, those respondents who respond to 3 indicators were considered as involved in birth preparedness and complication readiness. Adequate knowledge of danger signs was considered when the respondents respond to greater than or equal to five key danger signs during pregnancy, delivery and post-natal period, out of ten danger signs. The respondents who knew greater than or equal to two components of birth preparedness and complication readiness out of four components were considered as they had adequate knowledge of birth preparedness and complication readiness. Data was checked for completeness and consistency, coded, entered and cleaned with Epi-info version 7 and exported to SPSS (Statistical Package for Social Science) version 16 for analysis. The results were presented in the form of tables, and text using frequencies and summary statistics such as mean, standard deviation, and percentage to describe the study population in relation to relevant variables. The data were analyzed using logistic regression to determine the effect of various factors on the outcome variable and to control confounding. Crude and adjusted odds ratios with their 95% confidence intervals were calculated. Ethical clearance was obtained from Ethical Review Committee of University of Gondar, Department of Midwifery. A formal letter of cooperation was written to Debre Berhan town health department and each selected kebele administrations. Voluntary verbal consent was obtained from each study participant.

## Results

**Socio-demographic characteristics of study participants:** a total of 803 of husbands whose wives were pregnant or had children less than one year old were included in the study with a 95% response rate. The median age of the respondents was 33 with inter quartile range of nine. Majority of the respondents were Amhara (88.2%) by ethnicity. Regarding their educational status, majority had attended tertiary and above educational level (44.7%). More than half of respondents were not employed (58.5%) and the median income of the respondents was 2800 ETB (Ethiopian Birr) per month with inter quartile range of 1501ETB. Almost all of the respondents had monogamous marriage (99.9%) (Table 1).

**Table 1:** Socio-demographic characteristics of study, North Eastern Ethiopia, June 2014 (n = 803)

Variables	Frequency	Percent
**Age in years**		
20-29	224	27.9
30-39	409	50.9
40-49	158	19.7
≥50	12	1.5
**Ethnicity**		
Amhara	708	88.2
Tigre	29	3.6
Omoro	62	7.7
Other^1^	4	0.5
**Education**		
Can’t read and wright	79	908
Primary	100	12.5
Secondary	265	33.0
Tertiary	359	44.7
**Occupationnal status**		
Not employed	470	59.5
Employed	333	41.5
**Income**		
≤1000	54	6.7
1001-2000	287	35.7
2001-3000	216	26.9
≥3001	246	30.6
**Number of children**		
0	86	10.7
1 or 2	502	62.5
3 or 4	198	22.2
5+	37	4.6

1 Gurage, Wolaita

**Figure 1:**
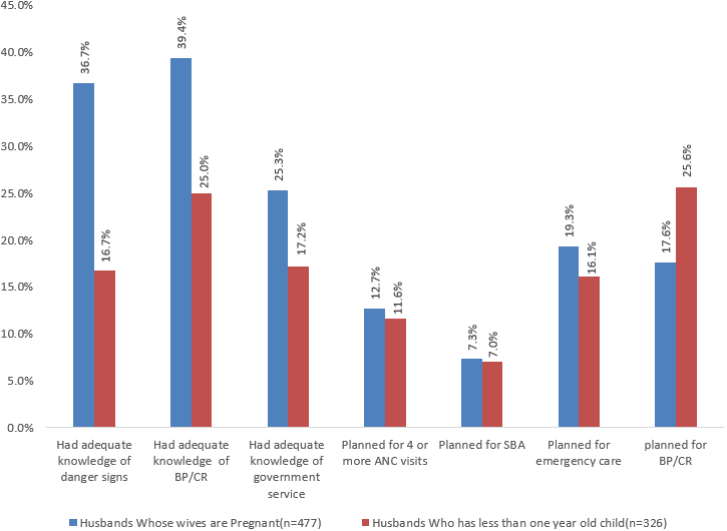
Distribution of birth preparedness and complication readiness indicators among study participants in Debre Berhan town, North Shoa Zone, North Eastern Ethiopia, June, 2014 (n = 803)

**Male involvement in birth preparedness and complication readiness:** the study found out that 51.4% (95% CI: 41.6%, 61.2%) of males involved in BP/CR. From the components, majority of husbands had adequate knowledge on BP/CR (64.4%) followed by who had adequate knowledge on danger signs (53.4%); and small number of husbands planned to have SBA (14.3%). Husbands whose wives were pregnant better met the components than husbands who had children less than one year old. For instance, with regard to knowledge on danger sign, 36% of husbands whose wives were pregnant, but 16.7% of husbands who had children less than one year old had adequate knowledge on danger sign (Figure 1).

**Factors associated with male involvement in birth preparedness and complication readiness:** in bivariate analysis with p-value of less than 0.05, the factors found to be significantly associated with male involvement were: education, occupational status, number of children, knowledge on danger signs, knowledge on BP/CR, attitude, practice, cultural belief and gender role. From variables found to be significant in bivariate analysis; education, number of children, knowledge on danger signs, knowledge on BP/CR and attitude were found to be significantly associated with male involvement in BP/CR in multivariate analysis. Those husbands with tertiary education level were about 4 times (AOR = 4.37 95% CI: 2.10, 9.13) more likely to be involved in BP/CR than those who can’t read and write. With respect to the number of children, the study finding showed that those who had 3 or 4 children were 5 times (AOR=5.19 95% CI: 2.44,11.03) more likely to be involved in BP/CR than those who had no child. Husbands who had adequate knowledge on danger sign were about 8 times more likely to be involved than those who had no adequate knowledge on danger signs of pregnancy, childbirth and post natal period (AOR = 7.71 95% CI: (5.15, 11.5)). Similarly, those husbands who had adequate knowledge on BP/CR were about 12 times (AOR = 11.98 95% CI: 7.73, 18.56) more likely to be involved in BP/CR than those who had no adequate knowledge. Husbands who had good attitude towards male involvement in BP/CR were about 2 times more likely to be involved in BP/CR than those who had poor attitude (AOR = 2.23 95% CI: 1.41,3.51) (Table 2).

**Table 2:** Bivariate and multivariate analyses of factors associated with male involvement in BP/CR in North Shao zone, Debre Berhan town, June, 2014 (n = 803)

Variables	Male Involvement		COR(95%CI)	AOR(95%CI)	P-Value
	Involved Number (%)	Not Involved Number (%)			
**Educational Status**					
Unable to read and write	22(27.8)	57(72.2)	1	1	
Primary	26(26.0)	74 (74.0)	0.91 (.47, 1.77)	0.78 (0.33, 1.85)	0.57
Secondary	100(37.7)	165 (62.3)	1.57(.91,2.72)	1.13(0.54, 2.35)	0.75
Tertiary	265 (73.8)	94 (26.2)	**7.30(4.23,12.60)**	**4.37(2.10, 9.13)**	0.001
**Occupational status**					
Not employed	173 (36.8)	297(63.2)	1	*	
Employed	240 (72.2)	93 (27.8)	4.43(3.27, 6.00)		
**Number of children**					
0	29(33.7)	57(66.3)	1	1	
1or 2	264(52.6)	238(47.4)	2.18	**2.52(1.30, 4.87)**	0.01
3 or 4	111(62.4)	67(37.6)	3.26	**5.19(2.44, 11.03)**	0.001
5+	9 (24.3)	28 (75.7)	0.63	2.47(.78, 7.87)	0.12
**Knowledge on danger signs**					
Adequate	317(73.9)	112(26.1)	8.20(5.97,11.25)	**7.71(5.15, 11.54)**	0.001
Not adequate	96 (25.7)	278 (74.3)	1	1	
**Knowledge on BP/CR**					
Has adequate knowledge	355(68.7)	162(31.3)	8.61(6.11,12.14)	**11.98(7.73,18.56)**	0.001
Has no adequate knowledge	58(20.3)	228(79.7)	1	1	
**Experience on BPCR**					
Had experience	282(69.1)	126(30.9)	4.510(3.35,6.07)	*	
Had no experience	131(33.2)	264(66.8)	1		
**Cultural belief of Male involvement in BPCR**					
In support	317(46.8)	360(53.2)	3.634(2.35,5.62)	*	
Against	96(76.2)	30(23.8)	1		
**Attitude towards male involvement in BPCR**					
Good attitude	277(48.2)	298(51.8)	1.59(1.16,2.17)	**2.23(1.41,3.51)**	0.01
Poor attitude	136(59.6)	92(40.4)	1	**1**	
**Gender role**					
Role encourages	313(46.4)	362(53.6)	4.131**(2.65,6.45)**	*	
Role discourages	100(78.1)	28 (21.9)	1		

1-Referent category; P- value set at less than 0.05 for bivariate and multivariate analysis;

* = not significant in backward stepwise logistic regression

## Discussion

The current study found out that the male involvement in BP/CR was 51.4%. This result is higher than the result found in Tigray region Enderta woreda which was 26.90% [10].This difference may be due to the difference in socio cultural differences and the socio demographic characteristics of study participants. For instance as a study done in Endarta woreda, Tigray region, Ethiopia only 21% of husbands had formal education and 17% of husbands had income level of more than 1000ETB which is much lower than current study [10]. The current study finding was comparable with analysis of DHS data in selected African countries (45.7%) [11]; but different from another studies done in peri-urban Gulu district, Northern Uganda (65.4%), India (56.5%) and Nepal (82.6%) respectively [12-14]. This difference might be due to variation in data collection tools, difference in socio political and socio economic status.

Husbands who attended tertiary education were more likely to be involved in BP/CR than those who can’t read and write. This is consistent with the studies done in different African countries and other countries such as India, Guatemala and Nepal [13-19]. This might be due to the fact that educated husbands may be more open toward health care service and aware of the benefits of skilled attendance and more able to talk with health workers and seek appropriate care for their wives [20]. Those husbands who had children were more likely to be involved in BP/CR than those who had no child. This is inconsistent with research done in Kenya [14]. This might be associated with fertility preference of Ethiopian males. As the number of children increases from no to three to four children; joint decision on husband earnings, women’s participation in their own health care and husbands’ participation in maternal health care increases [21]. Another possible explanation could be; when the number of children increases the bonding between wife and husband may increase and that might force husbands to involve in BP/CR actively. In African communities, children secure marital ties, offer social security, strength social interaction, secure rights of property and inheritance, maintaining the family lineage, and satisfy emotional needs [22].

Knowledge of danger sign is also one of the factors that have an effect on male involvement. Those husbands who had adequate knowledge on danger sign were more likely to be involved than those who had no adequate knowledge on danger signs of pregnancy, childbirth and post-natal period. This result is similar with the findings from the researches done in Harari public health institutions, Eastern Ethiopia and other countries such as Nigeria and Nepal [17, 23, 24]. This may imply having knowledge of danger signs to encourage husbands to seek health service care and make them to involve in BP/CR; as improving the awareness and skills of husbands could make them to be more involved in their wives’ health care and may make them to accompany their wives at the health facility [14]. When men become familiar with the danger signs of pregnancy and childbirth, they may become gate keepers ensuring that their spouse gets proper attention in pregnancy related emergencies [25]. Furthermore; when men are able to identify danger signs, they facilitate women’s utilization of health care services especially in emergency situations [12, 26].

Knowledge of husbands on BP/CR has significant effect on their involvement in BP/CR. Those husbands who had adequate knowledge on BP/CR were more likely to be involved in BP/CR than those who had no adequate knowledge. This result is similar with research done in India [27]. This might be due to the fact that, having knowledge may enhance to participate in issues which are helpful for maternity care such as BP/CR, but the finding from this study was different from the cross sectional study done in Khairahani village development committee of Chitwan district. There was low participation of husbands in birth preparedness in those also who were knowledgeable about it [27]. This disparity could be due to sample size, socio economic and socio-cultural variation. On other hand, husbands who had good attitude towards male involvement in BP/CR were more likely to be involved in BP/CR than those who had poor attitude. This finding supports the evidence that women whose husbands had positive perception of the use of a skilled birth attendant had more likely hood to make use of SBA [19]. In addition, men’s positive gender attitude enhances maternal health care utilization because of increased participation of their male partners [27]. Possible explanation of this finding is when the husbands have positive attitude towards involving in maternal health care’s such as antenatal care (ANC), childbirth and prenatal care (PNC); their involvement in BP/CR may increase and if husbands perceive maternal health as a women’s concern, men are less involved in maternal health [28]. Furthermore, when husbands perceive as their main duty was to secure financial issue; but activities like accompanying spouses to health facility should be reserved to women; their involvement decreases [26, 29].

## Conclusion

Male involvement in birth preparedness and complication readiness was found to be low. Socio demographic factors (education and number of children); individual related factors (knowledge on danger signs, knowledge on birth preparedness and complication readiness and attitude) were significantly associated with male involvement in birth preparedness and complication readiness. Creating awareness on danger signs of pregnancy, birth preparedness and complication readiness both at community and institutional level were recommended in order to increase male involvement in birth preparedness and complication readiness.

### What is known about this topic

- Magnitude of male involvement in BPRC is low;
- Known factors for high male involvement BPRC was having good attitude towards birth preparedness and complication readiness.

### What this study adds

- Attending tertiary level of education;
- Having good knowledge on danger sign and birth preparedness and complication readiness.
